# Urgent call for better use of existing vaccines and development of new vaccines to tackle AMR

**Published:** 2022-08

**12 July 2022** - WHO today released the first-ever report on the pipeline of the vaccines currently in development to prevent infections caused by antimicrobial-resistant (AMR) bacterial pathogens. WHO’s analysis points to the need to accelerate trials for AMR related vaccines in late-stage development and maximise the use of existing vaccines.

The silent pandemic of antimicrobial resistance is of major growing public health concern. Resistant bacterial infections alone are associated with nearly 4.95 million deaths per year, with 1.27 million deaths directly attributed to AMR. But AMR is about more than bacterial infections. AMR occurs when bacteria, viruses, fungi, and parasites change over time and no longer respond to medicines. When an individual becomes infected with these microbes, the infection is said to be resistant to antimicrobial medicines. These infections are often difficult to treat.

Vaccines are powerful tools to prevent infections in the first place, and therefore have the potential to curb the spread of AMR infections. The AMR vaccine pipeline report aims to guide investments and research into feasible vaccines to mitigate AMR.

The analysis identifies sixty-one vaccine candidates in various stages of clinical development, including several in late stages of development to address diseases listed on the bacterial priority pathogens list, which WHO has prioritized for R&D. While the report describes these late-stage vaccine candidates as having a high development feasibility, the report cautions that most will not be available anytime soon.

“Preventing infections using vaccination reduces the use of antibiotics, which is one of the main drivers of AMR. Yet of the top six bacterial pathogens responsible for deaths due to AMR, only one, Pneumococcal disease (Streptococcus pneumoniae) has a vaccine” said Dr Hanan Balkhy, WHO Assistant Director-General, Antimicrobial Resistance. “Affordable and equitable access to life-saving vaccines such as those against pneumococcus, are urgently needed to save lives, and mitigate the rise of AMR” she added.

The report calls for equitable and global access to the vaccines that already exist, especially among populations that need them most in limited-resource settings. There are already vaccines available against four priority bacterial pathogens: pneumococcal disease (Streptococcus pneumoniae), Hib (Haemophilus influenzae type b) Tuberculosis (Mycobacterium tuberculosis) and Typhoid fever (Salmonella Typhi). Current Bacillus Calmette-Guérin (BCG) vaccines against tuberculosis (TB) do not adequately protect against TB and the development of more effective vaccines against TB should be accelerated. The remaining three vaccines are effective, and we need to increase the number of people receiving them to contribute to a reduction in the use of antibiotics and prevent further deaths.

Of significance in the global fight against AMR, the bacteria noted in the priority pathogens list pose a significant threat to public health precisely because of their resistance to antibiotics – but they currently have a very weak vaccine pipeline in terms of the number of candidates and feasibility. Vaccines against these pathogens are unlikely to be available in the short term, and alternative interventions should be pursued urgently to prevent resistant infections due to priority bacterial pathogens

“Disruptive approaches are needed to enrich the pipeline and accelerate vaccine development. The lessons from Covid 19 vaccine development and mRNA vaccines offer unique opportunities to explore for developing vaccines against bacteria’’ said Dr Haileyesus Getahun, WHO Director of AMR Global Coordination Department.

 The report examines some of the challenges facing vaccine innovation and development, including for pathogens associated with hospital-acquired infections (HAI). These include the difficulty in defining target population(s) among all admitted hospital patients; the cost and complexity of vaccine efficacy trials; and the lack of regulatory and/or policy precedent for vaccines against HAIs.

“Vaccine development is expensive, and scientifically challenging, and are associated with high failure rates. For successful candidates complex regulatory and manufacturing requirements often exist. We have to leverage the lessons of COVID vaccine development and speed up our search for vaccines to address AMR.” said Dr Kate O’Brien, Director Immunization, Vaccines and Biologicals Department at WHO.

Available from: https://www.who.int/news/item/12-07-2022-urgent-call-for-better-use-of-existing-vaccines-and-development-of-new-vaccines-to-tackle-amr

